# Interview with Zining Liu, recipient of the 2026 Epilepsia Open Prize for Basic Science Research

**DOI:** 10.1002/epi4.70322

**Published:** 2026-08-13

**Authors:** Merab Kokaia, Piero Perucca

**Affiliations:** ^1^ Faculty of Medicine, Epilepsy Center Lund University Lund Sweden; ^2^ Department of Medicine (Austin Health) The University of Melbourne Melbourne Victoria Australia; ^3^ Bladin‐Berkovic Comprehensive Epilepsy Program Austin Health Melbourne Victoria Australia

Zining Liu discusses the award‐winning research, its impact, and future directions.

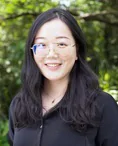

## 1 PLEASE TELL US ABOUT YOURSELF, INCLUDING YOUR EDUCATION, TRAINING, AND CURRENT POSITION

I am an early‐career postdoctoral research fellow specializing in animal models of epilepsy and preclinical research on cardiac function in epilepsy in the Department of Neuroscience at Monash University, Australia. I completed my PhD at Monash University from 2019 to 2023, supported by the Research Training Program Scholarship and the Monash Departmental Scholarship, with a thesis centered on cardiac dysfunction in epilepsy.

During my doctoral training, I worked to characterize structural, functional, and molecular cardiac abnormalities in temporal lobe epilepsy, with a particular interest in autonomic dysfunction and its contribution to long‐term morbidity and mortality. I have also worked on clinical studies of cardiac arrhythmias in people with chronic drug‐resistant epilepsy, including long‐term ECG monitoring and genotyping. Across these projects, I have become increasingly interested in how epilepsy should be understood not only as a disorder of recurrent seizures, but as a systemic condition with important comorbidities that may influence quality of life and survival.

Outside the laboratory, I enjoy mentoring students and early‐career researchers, and I value the collaborative side of science very much. I am also interested in scientific communication and in finding ways to make complex research questions accessible to both clinicians and patients.

## 2 HOW DID YOU BECOME INTERESTED IN CONDUCTING RESEARCH IN THIS FIELD?

I was first drawn to neuroscience and epilepsy because it is such a complex condition. It can be dramatic in its clinical presentation, but is also deeply heterogeneous in its causes, manifestations, and outcomes. As I learned more about epilepsy, I became increasingly interested in its comorbidities and its effects on peripheral systems. I became interested in the idea that recurrent seizures are not isolated events, but may gradually influence autonomic regulation, cardiac structure, and physiological resilience over time. Understanding those changes felt important not only from a mechanistic perspective, but also because they may be relevant to premature mortality and to improving long‐term care.

## 3 DESCRIBE THE QUESTION YOUR STUDY ADDRESSED AND HOW YOU APPROACHED IT

Our study asked whether epilepsy can cause structural and molecular changes in the heart, both soon after status epilepticus and later during chronic epilepsy. Cardiac abnormalities have been reported in people with epilepsy, but the mechanisms behind them are still unclear.

To study this, we used two rodent models of temporal lobe epilepsy: a kainic acid model in rats (KASE) and an electrical self‐sustained status epilepticus model in mice (SSSE). Using two different models allowed us to determine whether similar cardiac changes could be observed across species and different methods of inducing epilepsy.

We collected heart tissue at an early time point after SE and again during the chronic epileptic stage. We then examined fibrosis and measured the expression of key ion channels and exchangers involved in cardiac channelopathies. This approach enabled us to assess both structural remodeling and molecular changes over time.

## 4 WHAT WERE THE KEY FINDINGS, AND HOW DO YOU INTERPRET THEM?

The main finding was that both models showed clear evidence of cardiac alteration after epilepsy induction. We found increased fibrosis in the heart, as well as changes in the expression of several ion channels and exchangers that are important for normal cardiac conduction and excitability. Some changes appeared early, while others were still present during chronic epilepsy.

Although the exact pattern was not identical in every region or model, the overall message was consistent: the heart does not remain unaffected in epilepsy. Seizures appear to be associated with both structural remodeling and molecular changes in cardiac tissue.

I interpret these findings as further evidence that epilepsy is a systemic disorder, not only a disorder of recurrent seizures. The fact that similar cardiac changes were seen in two different models suggests that these abnormalities are likely related, directly or indirectly, to seizures themselves. These findings support the idea that cardiac health should be considered more carefully in epilepsy research and clinical management.

## 5 WHAT ARE THE NEXT STEPS FOR YOUR RESEARCH, AND WHAT ARE YOUR BROADER CAREER GOALS?

The next step in my research is to better understand the mechanisms that drive cardiac comorbidity in epilepsy and to identify targets for treatment or risk reduction. I am particularly interested in work that links preclinical models with clinical questions. This includes studying mechanisms in animal models while considering how those findings could inform monitoring, biomarker development, and patient care.

I also want to build on our earlier proof‐of‐concept work on cardioprotective strategies in epilepsy. In the long term, I hope this line of research will contribute to practical ways of improving cardiac management in people with epilepsy, especially those with severe or drug‐resistant disease.

My career goal is to develop an independent research program focused on epilepsy comorbidities, with a strong translational focus.

## 6 WHAT DOES RECEIVING THE EPILEPSIA OPEN PRIZE FOR THE BEST BASIC SCIENCE ARTICLE PUBLISHED IN 2025 MEAN TO YOU, YOUR TEAM, AND YOUR FUTURE WORK?

Receiving the Epilepsia Open Prize is a great honor. On a personal level, it is very encouraging to have this work recognized by the epilepsy research community. As an early‐career researcher, this kind of recognition gives me confidence to continue developing this area of research.

For our laboratory and institution, I see the award as recognition of collaborative and translational epilepsy research. This work was shaped by strong mentorship and by an environment that supports both experimental and clinically relevant questions.

For my future work, this prize helps create momentum. It strengthens my goal of building an independent research program in cardiac comorbidities in epilepsy and supports future fellowship and grant applications. More importantly, it helps highlight an area that deserves greater attention.

## CONFLICTS OF INTEREST STATEMENT

None of the authors has any conflicts of interest to disclose.

## Data Availability

Data sharing not applicable to this article as no datasets were generated or analysed during the current study.

